# MicroRNA-375 exacerbates knee osteoarthritis through repressing chondrocyte autophagy by targeting ATG2B

**DOI:** 10.18632/aging.103073

**Published:** 2020-04-26

**Authors:** Hongxing Li, Zhiling Li, Yigang Pi, Yang Chen, Lin Mei, Yong Luo, Jingping Xie, Xinzhan Mao

**Affiliations:** 1 Department of Orthopedics, The Second Xiangya Hospital of Central South University, Changsha 410011, Hunan, China; 2 Center of Health Management, The Central Hospital of Shaoyang, Shaoyang 422000, Hunan, China; 3 Department of Orthopedics, The Central Hospital of Shaoyang, Shaoyang 422000, Hunan, China

**Keywords:** osteoarthritis, chondrocytes, miR-375, autophagy

## Abstract

Objective: This study aimed to explore the underlying mechanism of miR-375 in exacerbating osteoarthritis (OA).

Results: MiR-375 expression were upregulated in OA cartilage tissues, whereas ATG2B expression was decreased. MiR-375 targeted ATG2B 3’ UTR and inhibited its expression in the chondrocytes, and then suppressed autophagy and promoted endoplasmic reticulum stress (ERs). The apoptosis rate of chondrocytes was increased after being transfected with miR-375 mimics. *In vivo* results further verified that inhibition of miR-375 could relieve OA-related symptoms.

Conclusion: miR-375 can inhibit the expression of ATG2B in chondrocytes, suppress autophagy and promote the ERs. It suggests that miR-375 could be considered to be a key therapy target for OA.

Methods: Differential expression analyses for mRNA and miRNA microarray datasets from ArrayExpress were performed. MiR-375 and ATG2B expressions in cartilage tissues were detected by qRT-PCR. Dual luciferase assay was applied to verify the targeting relationship between ATG2B and miR-375. *In vitro*, the role of miR-375 on chondrocyte autophagy and ERs was investigated by western blot and immunofluorescence. The apoptotic rate was quantified by flow cytometry. *In vivo*, OA mice model was established, HE and Safranin O and Fast Green staining, as well as the OARSI and modified Mankin scores, were applied to measure the OA cartilage damage severity.

## INTRODUCTION

Osteoarthritis (OA) is a disease with high morbidity, which impairs human health and social economy [1, 2]. It mainly afflicts the weight-bearing joints, such as the hips and knees, and causes physical disability. However, the precise pathogenesis of OA has not been detailed completely. According to the literature, there is few efficient treatments for OA except total joint arthroplasty for end-stage OA [3]. Therefore, it is crucial for prevention and treatment to systematically understand the mechanism underlying OA and find out a new approach to treat it effectively. Research showed that chondrocyte autophagy, as a self-protective mechanism, has been considered as a potential target for recuperating chondrocytes viability and then suppressing the progression of OA [4–6]. Cellular dysfunction and death often occur when the capacity of endoplasmic reticulum could not bear the protein folding under prolonged endoplasmic reticulum stress (ERs) [7, 8]. Hence, the occurrence of ERs would aggravate OA severity. The effects of autophagy and ERs on osteoarthritis remain to be further explored.

MicroRNAs (miRNAs) have been suggested to participate in regulating gene expression after transcription in OA [9, 10]. MiRNAs could suppress gene expression via targeting its 3’UTR region, which either blocks the translation process or induces cleavage [11]. These small regulators serve vital function in various biological processes [12]. Accumulating research has suggested that some miRNAs had regulatory effect in the formation and process of OA. For instance, D’Adamo et al*.* have unclosed that miR-155 inhibits autophagy in chondrocytes by regulating autophagy proteins expression [13]. MiR-375 was also found to be connected with cell autophagy, which could inhibit the autophagy activity of hepatocellular carcinoma under hypoxic conditions [14]. However, few researches have explored the role of miR-375 in OA.

As the main regulators in autophagy process, the expressions of the autophagy-related genes (ATGs) are usually up-regulated with a magnified autophagy activity [15]. Jin et al*.* revealed that Gcn4, Gat1, Gln3 and Sfl1 act on ATGs in autophagy process as transcriptional activators [16]. Besides, ERs were involved in autophagy. Tan et al. discovered that ERs induced apoptosis and autophagy while ATGs contributed to the regulation of autophagy [17]. As a member of the ATG family, ATG2B has the familiar function with ATG family. Previous studies have demonstrated the role of ATG2B in other diseases [15, 18, 19]. Nonetheless, the mechanism of ATG2B affected cell autophagy and apoptosis remains to be further studied, and its role in OA remains to be explored.

Animal models are of great importance in presenting underlying mechanisms of joint damage caused by OA. In addition, they also provide evidence for conceptive design in the progress of pharmacological and biological therapeutic [20]. These animal models were designed to reveal the different mechanisms through which stress results in OA progression as follow: the transection of the meniscus and/or ligaments [21, 22], the intra-articular administration of a chemical substance like papain, collagenase [23, 24]. In this study, destabilization of the medial meniscus (DMM) surgery induced OA model was established to analyze the role of miR-375 *in vivo*. We aimed to illuminate the potential role of miR-375 and ATG2B in OA.

## RESULTS

### Bioinformatic analysis

**Figure 1:**
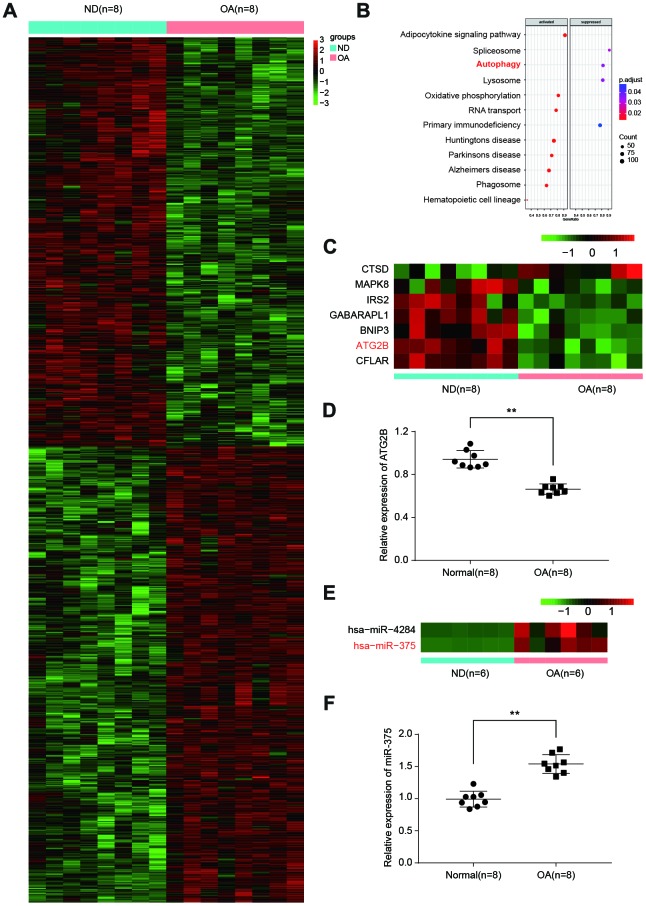
**Differential expressed mRNAs and miRNAs related to autophagy in OA.** (**A**) Heatmap of top differentially expressed mRNAs from 8 matched osteoarthritic and normal samples in osteoarthritis (OA) patients. (**B**) Significant function and pathways (*P*<0.05) based on KEGG database were analyzed using GSEA tool from upregulated and downregulated genes. Activated pathways were indicated in the left box, with suppressed pathways showing in the right box. (**C**) Heatmap depicting statistically significant (*P*<0.05) differentially expressed mRNAs related to autophagy. (**D**) Relative expression of ATG2B in osteoarthritic and normal samples was analyzed by qRT-PCR. (**E**) Heatmap depicting differentially expressed miRNAs related to autophagy from 6 matched osteoarthritic and normal samples. (**F**) Relative expression of miR-375 was analyzed by qRT-PCR.

DESeq2 analysis between OA and normal donors (ND) samples from ArrayExpress database identified mRNAs as differentially expressed with fold change (FD) >2 (log_2_fold>1) (Figure 1A). KEGG pathway analysis indicated that differentially expressed mRNAs were enriched in 12 biological pathways, including autophagy (Figure 1B). To categorize the altered autophagy-associated proteins in OA, we performed a similar differential analysis. As expected, autophagy-associated proteins were predominantly suppressed in OA, especially ATG2B (Figure 1C). By qRT-PCR, ATG2B was further confirmed to be downregulated in OA samples (Figure 1D). We applied the prediction algorithm of DIANA-mirPath (v3.0) to investigate the potential miRNAs targeting autophagy-related genes. The analyses found the increase of hsa-miR-375 and hsa-miR-4284 in OA (Figure 1E). MiR-375 was highly expressed in OA samples (Figure 1F), which was predicted to target ATG2B.

### MiR-375 targeting the 3’-UTR of ATG2B

**Figure 2:**
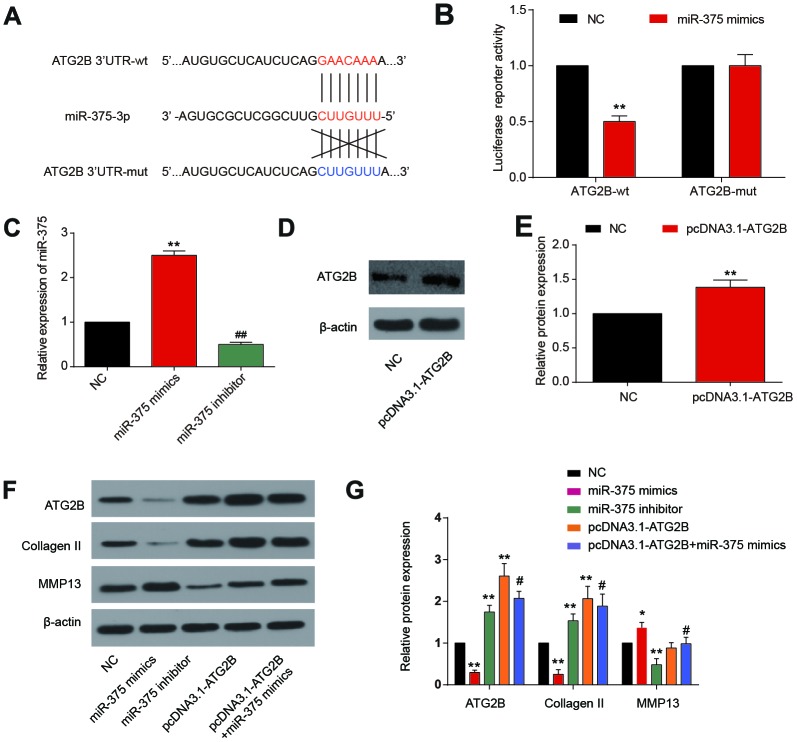
**MiR-375 targeting ATG2B.3’-UTR.** (**A**) miR-375 targeting the 3’-UTR of ATG2B predicted by TargetScan; (**B**) miR-375 mimics significantly reduced the wild 3’-UTR luciferase reporter activity of ATG2B; (**C**) OA chondrocytes were transfected with miRNA-375 mimics or inhibitor, and miRNA-375 expression was detected by qRT-PCR; (**D**, **E**) OA chondrocytes were transfected with pcDNA3.1-ATG2B, and ATG2B protein was detected by western blot, and the statistical results were presented; (**F**, **G**) protein expression of ATG2B, Collagen II and MMP13 were measured by western blot, and the statistical results were presented. * *P*<0.05, ** *P*<0.01, compared with NC group; #, *P*<0.05, compared with pcDNA3.1-ATG2B group.

Based on the TargetScan database, we predicted that miR-375 could target ATG2B 3’-UTR region (Figure 2A). MiR-375 mimics could significantly reduce the activity of wild 3’-UTR, without affecting the luciferase activities of the mutated 3’-UTR, indicating that miR-375 could directly bind to ATG2B 3’-UTR (Figure 2B). The transfection efficiency of miRNA-375 mimics and inhibitor in chondrocytes was tested by qRT-PCR 24h after transfection, as well as pcDNA3.1-ATG2B. In Figure 2C, miRNA-375 expression in transfected chondrocytes was significantly elevated by miRNA-375 mimics compared to NC (*P*<0.01). Meanwhile, pcDNA3.1-ATG2B could significantly increase the expression of ATG2B (Figure 2D, 2E). In addition, due to the relationship with anabolism and catabolism in OA pathological processes, the expressions of Collagen II and MMP13 were detected [25]. Collagen II expression was significantly decreased after MiR-375 mimics transfection, while MMP13 expression was remarkably up-regulated (Figure 2F, 2G). The influence of miR-375 mimics on Collagen II and MMP13 was partially reversed by pcDNA3.1-ATG2B.

### Effect of miR-375 and ATG2B on ERs and autophagy in chondrocytes

**Figure 3:**
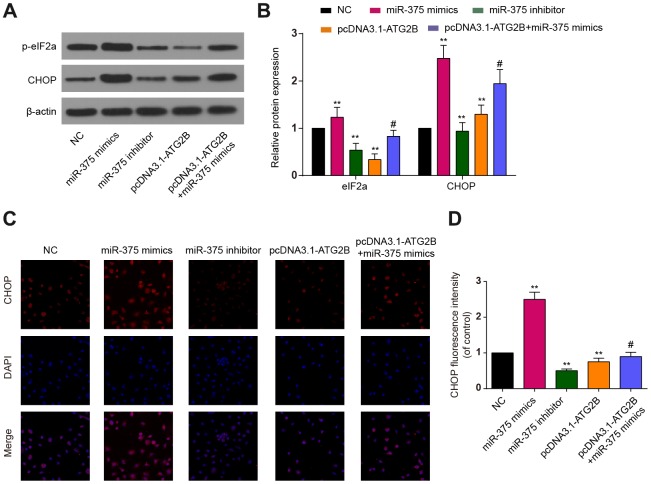
**The effects of miR-375 and ATG2B on ERs.** (**A**) Western blot of p-eIF2a and CHOP after infected with miR-375 mimics, miR-375 inhibitor, pcDNA3.1-ATG2B and pcDNA3.1-ATG2B plus miR-375 mimics in OA chondrocytes; (**B**) Qualitative analysis of p-eIF2a and CHOP, and the values were normalized to β-actin; (**C**, **D**) OA chondrocytes were double stained with CHOP (red) and DAPI (blue) and visualized by confocal microscopy after different treatment. ** *P*<0.01, compared with NC group; #, *P*<0.05, compared with pcDNA3.1-ATG2B group.

**Figure 4:**
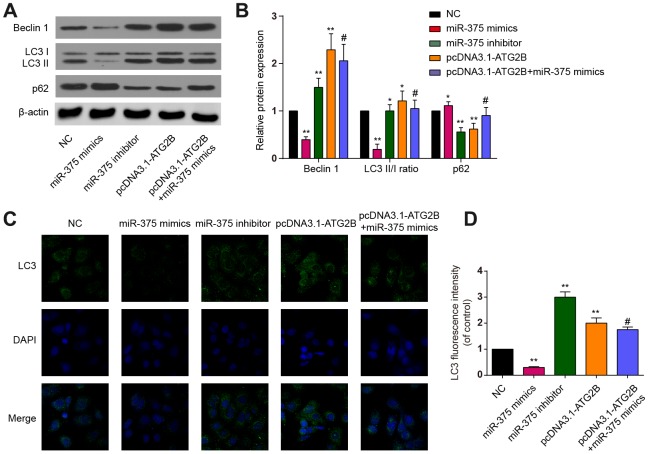
**The effects of miR-375 and ATG2B on autophagy.** (**A**) Western blot of Beclin 1, LC3 I/II and p62 after infected with miR-375 mimics, miR-375 inhibitor, pcDNA3.1-ATG2B and pcDNA3.1-ATG2B plus miR-375 mimics in OA chondrocytes; (**B**) Qualitative analysis of Beclin 1, LC3 I/II and p62, and the values were normalized to β-actin; (**C, D**) OA chondrocytes were double stained with LC3 (green) and DAPI (blue) and visualized by confocal microscopy after different treatment. * *P*<0.05, ** *P*<0.01, compared with NC group; # *P*<0.05, compared with pcDNA3.1-ATG2B group.

CHOP and p-eIF2a proteins, as ERs markers, were detected by western blot. Significant increases of p-eIF2a and CHOP expression were revealed after miR-375 mimics transfection, with the effects being reversed by pcDNA3.1-ATG2B (Figure 3A and 3B). Immunocytochemical analysis presented similar trends in CHOP expression in the OA chondrocytes (Figures 3C, 3D). Then, the influence of miR-375 and ATG2B on autophagy was explored. As indicated in Figure 4A, 4B, Beclin 1 and LC3 II expressions were significantly decreased, while P62 was increased by miR-375 mimics. Again, pcDNA3.1-ATG2B could reverse these effects. The immunofluorescence staining also showed that miR-375 mimics inhibited the LC3 II expression, but pcDNA3.1-ATG2B significantly increased the LC3 II expression (Figure 4C, 4D). Taken together, miR-375 overexpression inhibited autophagy and promoted ERs in chondrocytes.

### Effects of miR-375 and ATG2B on apoptosis of chondrocytes

**Figure 5:**
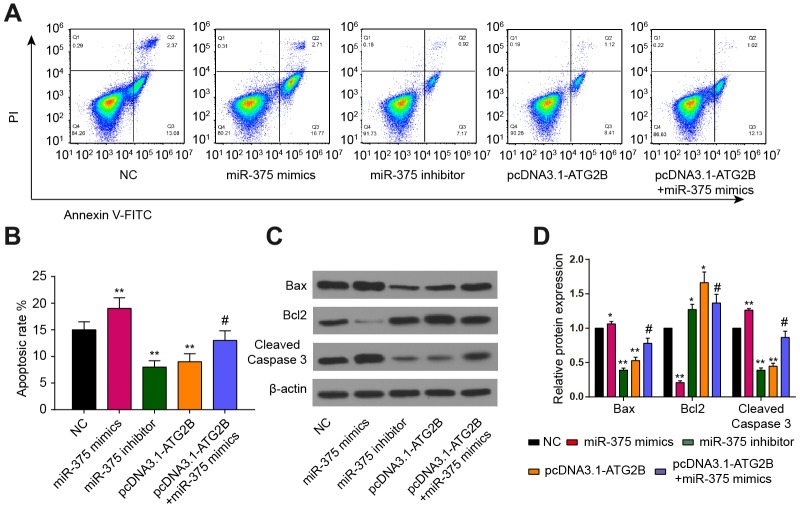
**Effects of miR-375 and ATG2B on apoptosis of chondrocytes.** (**A**, **B**) Effects of miR-375 and ATG2B on apoptosis of chondrocytes was detected by flow cytometry, and the statistical analysis were presented; (**C**, **D**) Protein expressions of Bax, Bcl2, cleaved caspase-3 in chondrocytes were measured by western blotting. * *P*<0.05, ** *P*<0.01, compared with NC group; # *P*<0.05, compared with pcDNA3.1-ATG2B group.

As shown in Figure 5A, apoptotic chondrocytes were remarkably increased after miR-375 mimics transfection, which could be partially reversed by pcDNA3.1-ATG2B.

The statistical results also confirmed that miR-375 mimics could induce chondrocytes apoptosis, while pcDNA3.1-ATG2B could protect chondrocytes from apoptosis (Figure 5B). In addition, miR-375 mimics significantly upregulated the expression of Bax, and cleaved Caspase-3, while repressed the expression of Bcl2, respectively (Figure 5C, 5D).

### MiR-375 antagomir alleviated DMM-induced OA

**Figure 6:**
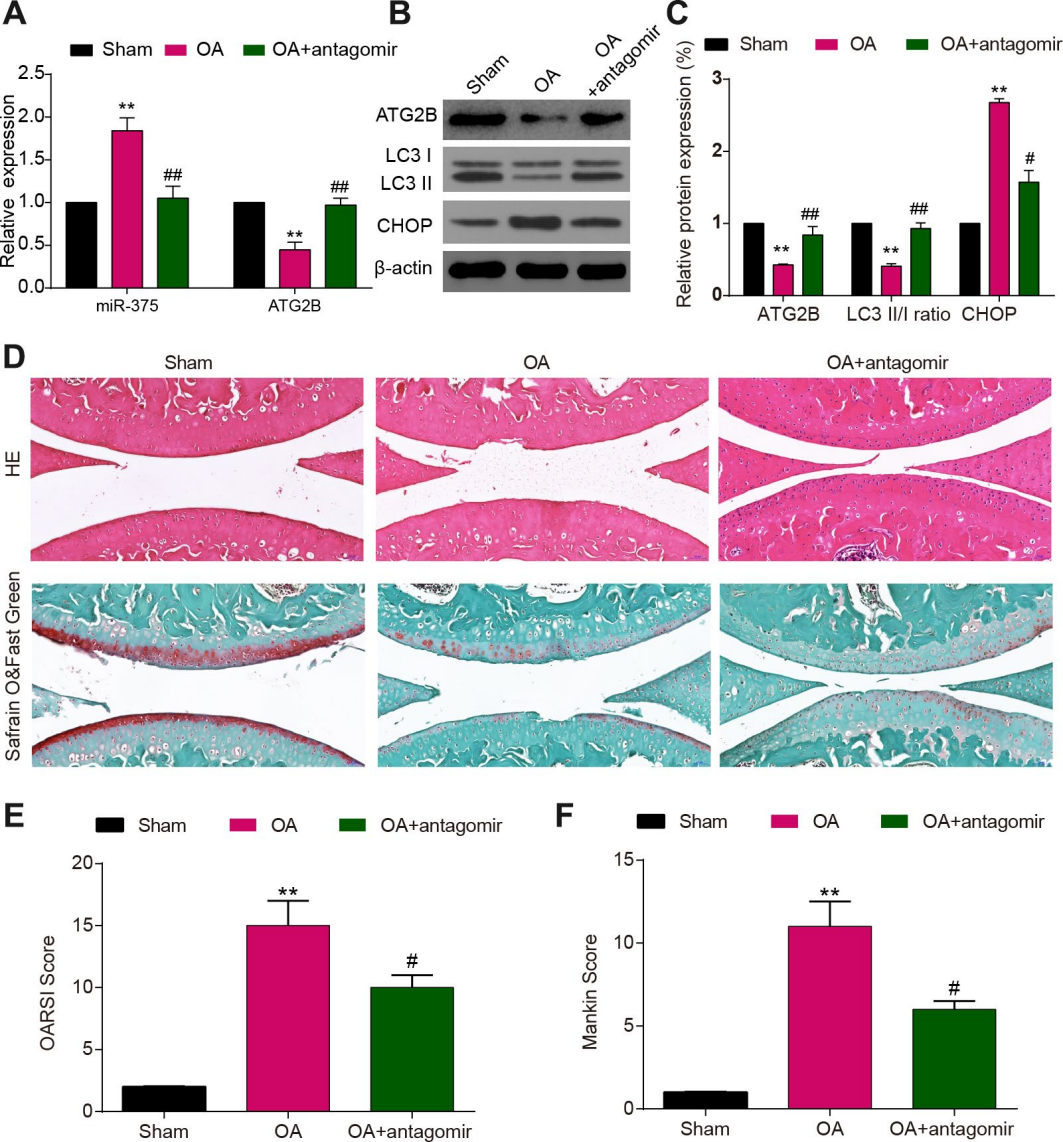
**MiR-375 antagomir alleviated DMM-induced OA.** (**A**) Down-regulated expression of miR-375 in chondrocytes after antagomir treatment and increased levels of ATG2B in DMM-injured OA knees. (**B**, **C**) Expression of ATG2B, LC3I/II, CHOP was detected by western blot. (**D**) Safranin O and Fast Green and HE stained sections of knee joints. (**E**, **F**) Cartilage destruction evaluated with the OARSI and Mankin scores. Scale bar: 50 μm. Data per group are expressed as mean ± SEM calculated from six mice. ** *P*<0.01, compared with sham group; # *P*<0.05, ## *P*<0.01, compared with OA group.

Specimens harvested from OA mice exhibited miR-375 over-expression, compared to the decline of ATG2B expression (Figure 6A). To evaluate the role of miR-375 on ATG2B and cartilage destruction, as well as autophagy and ERs of chondrocytes, injection of miR-375 antagomir was conducted at the knee joint cavity of the OA mice. The antagomir treatment significantly reduced the DMM-induced miR-375 expression in articular cartilage. As indicated in Figure 6B and 6C, the treatment of miR-375 antagomir dramatically up-regulated the expression of LC3 II in DMM-induced OA model, while the CHOP expression was suppressed by miR-375 antagomir. As the images shown in Figure 6D, articular cartilage damage was obviously observed in OA mice. By contrast, the miR-375 antagomir revealed to be beneficial for articular cartilage repairment. The OARSI and modified Mankin scores were applied to measure the OA cartilage damage severity (Figure 6E, 6F), and the results indicated that miR-375 antagomir remarkably alleviated cartilage damage of OA mice.

## DISCUSSION

In the current research, we analyzed the differentially expressed mRNAs and miRNAs between OA and normal cartilage tissues by analyzing microarray datasets of mRNAs and miRNAs in OA from ArrayExpress. In human samples, we discovered that miR-375 was over-expressed in OA, while ATG2B was conspicuously down-regulated in pathological OA articular cartilage tissues. *In vitro*, miR-375 inhibited autophagy and enhanced ERs of chondrocytes by suppressing the expression of ATG2B. Simultaneously, apoptosis of chondrocytes was promoted by miR-375 mimics. Furthermore, OA mice model induced by DMM surgery in the right knee was established and verified the function of miR-375 on exacerbating OA.

MiR-375 was first transcribed in Min6 cell, and it maintained a high degree of conservatism in species evolution [26]. Researchers have found that miR-375 took part in the regulation of biological and pathologic activities through multiple molecular mechanisms, especially in cancer [27]. In this study, miR-375 expression in OA articular cartilage tissues was measured by qRT-PCR. In OA cartilage tissues, miR-375 expression was highly expressed. The result indicated that dysregulated miR-375 could affect OA progression.

At present, many studied had revealed kinds of miRNAs were associated with autophagy process [28]. Korkmaz et al*.* determined that miR-376b could inhibit ATG4C and BECN1, as well as mTOR signaling pathway, then controlled starvation [29]. In addition, Song et al*.* found miR-21 acted as a regulatory factor of *GAS5* in the pathogenesis of OA, and it also stimulated chondrocytes apoptosis and cut down the expression level of autophagic complex. The interaction between miR-21and *GAS5* was suspected to be responsible for inhibiting the autophagy reaction [30]. With the discovery of high expression of miR-375 in OA, a new possibility has been found. For the clinical treatment of OA, miR-375 might be treated as a new therapeutic target. Inhibition of miR-375 was demonstrated to reduce apoptosis and promote autophagy of chondrocytes, which might attenuate the progression of OA.

MiRNAs is considered as a key factor in determining the role of gene silencing after transcription. Hence, their function and regulating mechanism are vital for understanding the biology of OA process, and may clarify their role in OA pathophysiology [31]. MiRNAs could suppress gene expression by binding 3’-UTR of targeted genes. The roles of miRNAs could be contributed to the synergetic interaction with its targets since a miRNA could bind to hundreds of genes, hence involving in various biological processes [14, 32, 33]. In the current study, the results demonstrated that miR-375 could inhibit autophagy through ATG2B. MiR-375 remarkably inhibited ATG2B expression, and the luciferase reporter assay demonstrated that miR-375 could directly bind to ATG2B 3’-UTR. These results illuminated that ATG2B is a key autophagy-related gene inhibited by miR-375. We suspect that ATG2B overexpression could promote chondrocytes to undergo autophagy, and protect chondrocytes from over-reaction to metabolic stresses, like ERs. The inhibitory role of miR-375 on ATG2B could be a potential biologic suppressor for autophagy. However, the collaborative effects of miR-375 by targeting other genes might lead to the inhibition of autophagy and apoptosis related signals, suggesting a synergic involvement of miR-375 in autophagy and ERs.

Autophagy and apoptosis exerted vital impacts on chondrocytes apoptosis in OA pathogenesis. As we all known, autophagy was an intrinsic and protective role in chondrocytes, which was correlated to OA and age-related loss in cartilage tissues [34]. ERs and autophagy are correlated in maintaining cellular homeostasis through a well-orchestrated mechanism [35, 36]. All is known that autophagy regulates cellular stress responses, ERs mediated by autophagy ensures ER protein homeostasis [37, 38]. The interaction between autophagy and ERs activation could influence the equilibrium among autophagy, apoptosis and ERs signals. Hence, we suspected that ATG2B overexpression could block ERs as soon as autophagy activation, which plays a protective role in inhibiting ERs.

Our study demonstrated miR-375 accelerated the progression of OA. Besides, by detecting autophagy monitoring proteins Beclin 1 and LC3 II, and p62, we found that miR-375 would inhibit autophagy process. Increasing studied indicated that ERs, a rising degree or duration of stress could contribute to apoptosis mechanism and pro-survival functions of autophagy process [39]. Recent studies showed that ERs was capable to promote or suppress autophagy [37, 38]. However, the stress-related mechanisms that regulate the transition switch between autophagy induction and inhibition are still fuzzy [40]. Previous research suggested that the ERs conditions were led by an accumulation of misfolded or unfolded proteins. Specific roles of miR-375/ATG2B in ERs process were still unclear and need further exploration. Therefore, further exploration of the complexity of the ERs response is worthy for development in novel therapeutic methods. In the current study, we discovered that ERs monitoring proteins, especially CHOP, were up-regulated in miR-375 mimics groups and the effect was offset by the treatment of pcDNA3.1-ATG2B. Besides, we tested the expression levels of apoptosis monitoring proteins, including Bax, Bcl2, and cleaved Caspase-3, which demonstrated that miR-375 mimics would promote apoptosis process. According to the research conclusion of targeting ERs induced apoptosis [41], our results have been validated. Hence, we concluded that ERs induced apoptosis might be related to miR-375/ATG2B regulatory network.

However, the effect of miR-375 on autophagy and ERs is still not clear, and it needs to be fully explained through reasonable experiments. In addition, this study doesn’t thoroughly explore the biological mechanism of ATG2B and miR-375 in OA chondrocytes ERs and autophagy. Further studies are needed in the future to illuminate the detailed mechanism. Furthermore, a more comprehensive regulatory network needs to be built to explore the OA pathological mechanism.

## CONCLUSIONS

In summary, we had provided an evidence of miR-375 deteriorating OA progression by suppressing autophagy and accelerating ERs process through regulating ATG2B. Furthermore, we set up an OA model to explore the effect of miR-375 *in vivo*. Therefore, miR-375 could be a potential target for OA treatment.

## MATERIALS AND METHODS

### Clinical samples

Articular cartilage tissues were isolated from eight knee OA patients at end-stage receiving total knee arthroplasties, and their clinical characteristics were listed in Supplementary Table 1. Cartilage from paired osteochondral samples were isolated from the intact PLC (posterior lateral condyle) and the damaged DMC (distal medial condyle). Ethical approval (2019141) was obtained from the Institutional Review Board of the Second Xiangya Hospital of Central South University. Written informed consent was obtained.

### Bioinformatic analysis

RNA-seq data for mRNA (E-MTAB-4304) and miRNA (E-MTAB-5715) are obtained in the ArrayExpress database (https://www.ebi.ac.uk/arrayexpress/) [42, 43]. Cartilage from 8 paired osteochondral samples were available for mRNAs screening, while cartilage from 6 matched osteoarthritic and normal samples were used for miRNAs screening. Analysis of the microarray data were carried out by using R software (ver. 3.4.1). Differential expression analysis was performed using DESeq2 package. Heatmaps were generated with log2 transformed and normalized counts using the pheatmap function. Biochemical and cellular pathways were clustered by employing Gene Set Enrichment Analysis (GSEA) tool. Based on the Kyoto Encyclopedia of Genes and Genomes (KEGG) database, GSEA identified the OA-related signaling pathways that were most significant to the data set (–log2[P value]>2.0). The GSEA analysis results were visualized using "ggplot2" package. To identify molecular pathways influenced by potential miRNAs, DIANA-mirPath (v3.0) was use to perform an enrichment analysis of specific miR targets, comparing each set of miR targets to genes and genomes pathways denoted by KEGG database. Potential targets of miR-375 was predicted by the TargetScan database.

### DMM-induced OA model

Experimental protocols for C57BL/6J mice were approved by the Animal Research Committee of the Central South University (2018sydw0015). Eighteen male mice (8 weeks) were obtained from Hunan SJA Laboratory Animal Co., Ltd (Changsha, China). The mice were separated randomly into three groups with six mice each: Sham group, OA group, and OA + miR-375 antagomir group. OA model in the right knee was established via DMM surgery, while the control group received sham non-injurious surgery using aseptic surgical procedures [44]. One week after surgery, intra-articular injection of antagomir-375 was given to the mice in antagomir group for 3 weeks (once a week). After 4 or 8 weeks, the mice were sacrificed and the keen joints were isolated for further experiments. For western blot and qPCR analysis, the cartilage tissues without subchondral bone were isolated.

### Histomorphology staining

For morphological analysis, the tissue sections were embedded in paraffin. Serial tissue sections at thickness of 5-μm in mid-sagittal were collected, and were stained with safranin O and hematoxylin and eosin (HE) to identify pathological features and proteoglycan content in the articular cartilage. The severity of OA articular cartilage degeneration was quantified by the Osteoarthritis Research Society International (OARSI) scores and modified Mankin scales [45, 46].

### Chondrocytes isolation

Tissues were cleaned by sterile phosphate buffer solution (PBS). The diced cartilage was digested overnight and then incubated in 10% fetal bovine serum (FBS) and high-glucose Dulbecco’s modified eagle medium (DMEM). The cell suspension was centrifuged for 5 min after being filtrated in a cell strainer. Primary chondrocytes were collected and cultured in high glucose DMEM and 10% FBS. First passage of primary chondrocytes was used for the experiments.

### Cell transfection

Chondrocytes were seeded in 96-well plates with 1×10^4^ cells/well and then incubated for about 24h. Transfections of miRNA-375 mimics (10 nM, Qiagen, Germantown, USA) or the corresponding miRNA-375 inhibitor (10 nM, Qiagen, Germantown, USA) were conducted via Lipofectamine RNAiMAX (Invitrogen, Carlsbad, USA). The transfection efficiency was verified 48h later, before performing further experiments. pcDNA3.1-ATG2B was also transfected into chondrocytes for 24h by Lipofectamine RNAiMAX (Invitrogen, Carlsbad, USA).

### Luciferase reporter assay

The wild-type and mutant-type sequences of the ATG2B 3’-UTR were constructed into luciferase receptor vectors, respectively. Luciferase reporter vector were transfected into chondrocytes followed by miR-375-3p mimics. Dual Luciferase Detection Kits (Promega) were used to quantify the luciferase activities after being normalized with the activity of Renilla luciferase.

### qRT-PCR

As for qRT-PCR of mRNA, TaqTM II Kit and PrimeScript RT reagent Kit (Takara, Japan) were used. β-actin was applied to be an internal reference. As for RT-PCR of miR-375, MicroRNA Isolation Kits (Biochain, USA), miRNA Fist-Strand cDNA Synthesis Kit and qPCR Kit (GeneCopoeia, America) were used with U6 as the internal standard. The 2^−ΔΔCt^ method was applied to measure the relative expression [47]. The primer sequences are presented in Supplementary Table 2.

### Western blot

RIPA (Beyotime, China) was used to harvest total protein of chondrocytes from cell lysates. Sodium dodecyl sulfate-polyacrylamide gel electrophoresis (SDS-PAGE) was applied for separating protein, and then protein was transferred to polyvinylidene difluoride (PVDF) membrane. Primary antibodies incubated for 24h, and then secondary antibody incubated in IgG conjugated horseradish peroxidase (HRP) for another 2h. Enhanced chemiluminescence (ECL) kit was applied to detect light-emitting signal. The primary antibodies were used for ATG2B, Beclin 1, LC3, p62, Collagen II, MMP13, CHOP, p-eIF2a, Bax, Bcl-2, Caspase 3, and β-actin (BOSTER, China).

### Chondrocytes apoptosis

The number of apoptotic chondrocytes accounting for a total of 10^5^ cells was quantified by flow cytometry with fluorescein isothiocyanate (FITC)-labeled Annexin V and propidium iodide (PI) (Enzo Life Sciences Inc., Farmingdale, NY).

### Immunofluorescence staining

Chondrocytes were fixed in paraformaldehyde and blocked with bovine serum albumin after being rinsed in PBS. Primary antibodies of CHOP or LC-3 incubated overnight, then HRP-conjugated goat anti-rabbit IgG incubated for 1h and DAPI labeled for 5mins. Fields were acquired by using fluorescence microscopy (Nikon, Japan), and Image J (Bethesda, USA) was used for fluorescence intensity quantification. Cells Positive chondrocytes for ERs or autophagy were defined as CHOP or LC3 labeled cells.

### Statistical analysis

Each experiment was conducted in triplicate, with data being presented as means ± standard deviation (SD). Statistical analyses were conducted using GraphPad Prism 5.0 (GraphPad Software, USA). Independent-sample student’s *t* test were used for comparison between two groups, while one-way analysis of variance (ANOVA) with post hoc test was used for multiple group comparisons. *P* < 0.05 was considered to be significant.

### Ethical approval

All procedures performed in studies involving human and animals were in accordance with the ethical requirements of The Second Xiangya Hospital of Central South University.

### Informed consent

Informed consent was obtained from all participants.

### Data availability statement

The data are available from the corresponding author upon reasonable request.

## Supplementary Material

Supplementary Tables
