# Supplementary material for: MicroRNA-375 exacerbates knee osteoarthritis through repressing chondrocyte autophagy by targeting ATG2B
Source: Aging (Albany NY). 2020 Apr 26;12(8):7248–61. doi: 10.18632/aging.103073 (PMC7202526; doi:10.18632/aging.103073)
Supplement: Supplementary Tables [file aging-12-103073-s001..pdf]

SUPPLEMENTARY TABLES

Supplementary Table 1. Clinical characteristics of osteoarthritis patients.

Samples	Disease	Age (year)	Gender	Modified Mankin score
Patient_1	osteoarthritis of the knee	63	male	20
Patient_2	osteoarthritis of the knee	65	male	17
Patient_3	osteoarthritis of the knee	70	female	18
Patient_4	osteoarthritis of the knee	72	female	20
Patient_5	osteoarthritis of the knee	66	male	18
Patient_6	osteoarthritis of the knee	65	male	19
Patient_7	osteoarthritis of the knee	69	female	17
Patient_8	osteoarthritis of the knee	71	male	19

Supplementary Table 2. Oligonucleotide sequences and RT-qPCR primers.

**Oligonucleotide sequences and RT-qPCR primers for human specimens**	
miR-375-3p	UUUGUUCGUUCGGCUCGCGUGA
U6	GTGCTCGCTTCGGCAGCACATATACTAAAATTGGAACGATACAGAGAAGATTAGC ATGGCCCCTGCGCAAGGATGACACGCAAATTCGTGAAGCGTTCCATATTTT
Atg2b	Forward: 5'-AACTGCTGACGAATCCTCAGG-3'<br>Reverse: 5'-GGGGTTCCAGCTAGGTGAGA-3'
β-actin	Forward: 5'-GTAACCCGTTGAACCCCATT-3'<br>Reverse: 5'-CCATCCAATCGGTAGTAGCG-3'
**Oligonucleotide sequences and RT-qPCR primers for mouse specimens**	
miR-375-3p	UUUGUUCGUUCGGCUCGCGUGA
U6	GTGCTCGCTTCGGCAGCACATATACTAAAATTGGAACGATACAGAGAAGATTAGC ATGGCCCCTGCGCAAGGATGACACGCAAATTCGTGAAGCGTTCCATATTTT
Atg2b	Forward: 5'-TGCCAGAGGTGTTTGTTGGT-3'<br>Reverse: 5'-TTGGCCAAGGGAAGTGGTTT-3'
β-actin	Forward: 5'-GTAACCCGTTGAACCCCATT-3'<br>Reverse: 5'-CCATCCAATCGGTAGTAGCG-3'
